# Reduction of Friction Using Microabrasive Air Jet Machining

**DOI:** 10.3390/mi14030649

**Published:** 2023-03-13

**Authors:** Sungcheul Lee, Soochang Choi, Hyeon Hwa Lee, Seung-Kook Ro

**Affiliations:** 1 Department of Ultra-Precision Machines and Systems, Korea Institute of Machinery & Material, 156, Gajeongbuk-ro, Yuseong-gu, Daejeon 34103, Republic of Korea; scchoi@pnudrone.com (S.C.); cniz@kimm.re.kr (S.-K.R.); 2 Molding & Metal Forming R&D Department, Korea Institute of Industrial Technology, Bucheon 14441, Republic of Korea; vikar02@kitech.re.kr

**Keywords:** smart surface texture, reduction of friction, micro abrasive air jet, micro-AAJ

## Abstract

In this paper, we introduce a microabrasive air jet (micro-AAJ) machining system and describe machining results obtained while using it. Our research activities have focused on the development of micro-AAJ machining methods for friction reduction without requiring a mask. Through this study, we want to show that such processing is possible without a mask when using the AAJ method. In this paper, a micro-AAJ machining system is introduced, and the processing results are described. By comparing the friction coefficient of texturing under various conditions, the relative relationship between the surface shape and the friction coefficient can be identified. We investigated the effect of the relationship between rotating velocity, traverse speed, density of the patterned area and injection pressure on friction. The friction is decreased with a low traverse speed and high-density patterned area under high-speed driving conditions, as verified by experiments using a friction test system.

## 1. Introduction

There have been growing demands for the development of energy-saving technologies. One aspect of improving energy efficiency is friction reduction on bearing surfaces, and one of the most obvious methods to achieve this is to reduce the surface roughness. To achieve this, additional processing steps are required, such as lapping of the surface. Surface texturing is an alternative approach for applications involving sliding-contact elements. The presence of shallow holes on bearing surfaces may act as fluid reservoirs, helping to retain a lubricating film between the parts. In addition, surface texturing may aid in wear prevention in start/stop automotive engine technology by retaining lubricant in the holes, thereby maintaining hydrodynamic tribological conditions.

Surface texturing can be achieved using techniques such as photolithography [1], electron beam lithography [2], or radio-frequency magnetron sputtering [3]. Takigawa [4] utilized a high-voltage electron beam lithography method to form 0.75 µm lines on PMMA and reported that using a weak developer such as isoamyl acetate (IAA) improves the dimensional accuracy of resist patterns drawn on uneven surfaces. Dubey [5] reviewed modeling and optimization techniques to determine optimal laser beam cutting conditions, in addition to reviewing laser beam machining (LBM) fields of various materials and shapes. He described that LBM is suitable for precision machining such as microholes with a high aspect ratio of 20 or more and a very small diameter of up to 5 µm.

In addition, technologies employing electric discharge can be used, including electrical discharge machining (EDM), electrochemical machining (ECM), and wire electrical discharge machining (WEDM). Byun [6] machined dimples with a diameter of 300 µm and a depth of 5 µm at intervals of 1 mm on an AISI 440C specimen to achieve a density of 5% using the micro-ECM method at a speed of 20 mm/s or less compared to a surface without dimples. It was reported that the coefficient of friction was improved. In addition, it was explained that the ECM method does not leave thermal or mechanical damage after pattern processing and is simpler than the photolithography process, which has advantages in micropattern processing. Oh [7] proposed the concept of electrically assisted indentation for surface texturing and studied the effect of current on diamond tool indentation using a Vickers hardness tester. His proposed process is a surface plastic deformation process of metals such as indentation and is applicable to functional surface texturing and marking. Ho [8] explained the need for research on the improvement of metal removal efficiency and process control of electrical discharge machining (EDM) and reported that the EDM method is suitable for complex micro-scale machining. Bhattacharyya [9] proposed a design for the development of a setup of an electrochemical microscopic recruitment (EMM) system with a tool diameter of about 150–200 µm, which was a challenge in the field of micromachining, especially in the machining of microparts in the electronics and precision industries. Chang [10] reported on an energy-enhanced (ee) microelectric discharge machining (EDM) system for the fabrication of nanotungsten (nano-W) colloids applicable to surface texturing processing. The nano-W colloid produced by the ee micro-EDM system has a particle size of about 11–12 nm, and this technology was proposed as an advanced technology capable of producing high-quality nano-W colloids. Ho [11] provided a review of studies involving optimization with monitoring and control of wire electrical discharge machining (WEDM) processes. In the review, he introduced the WEDM process as an unconventional material removal process that uses thin, continuously moving wire electrodes with a diameter of 0.05 to 0.3 mm, enabling machining of very small corner radii. 

**Figure 1:**
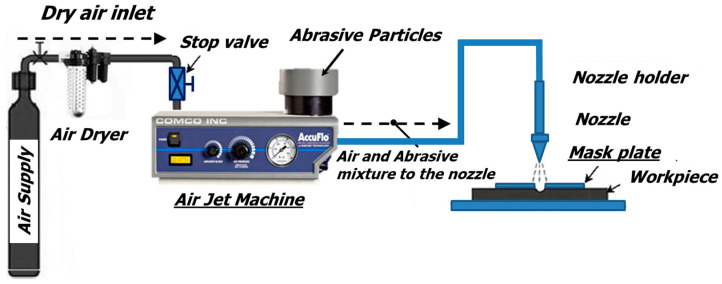
Typical AAJ machining system [18].

Here, we report a new manufacturing method for friction reduction using microabrasive air jet (micro-AAJ) machining. Abrasive air jet machining employs compressed air to accelerate a jet of high-speed particles to mechanically machine features, including micro-channels and microholes, into glass, metal, or polymer substrates for use in microfluidics, microelectromechanical systems (MEMS), and optoelectronic device fabrication. Because the jet spot diameter is typically in the order of 5 mm and the microfeatures to be machined typically have dimensions of 100–1000 µm, patterned openings in erosion-resistant masks are used as stencils to define the features, as shown in Figure 1. Shafiei [12] developed a computer simulation capable of predicting the shape and size of erosion profiles in abrasive jet machining processes. It was explained that this technology has the advantage of improving the process because the depth and shape of erosion can be predicted in an abrasive jet micromachining (AJM) operation using a mask. Boonkkamp [13] proposed a mathematical model for abrasive machining of holes in glass plates mounted on state-of-the-art flat-screen televisions. The proposed model includes first-order nonlinear partial differential equations for the location of the mask and glass plate, erosion of the mask by powder explosion, and displacement of the glass surface in order to precisely process the hole at an exact location. Dehnadfar [14] experimentally predicted the distribution of particle velocity and mass flux through a mask hole using a high-speed laser shadowgraphy technique in AJM. The mask opening width was 500–600 µm, with a thickness of 910 µm, and the mass flow distribution and velocity distribution of abrasive particles with an average size of 24–57 µm were analyzed. Saragih [15] presented the implementation of a thick SU-8 layer as a mask in the microabrasive jet machining (μ-AJM) process. He realized an arc-shaped microchannel with an aspect ratio of 0.33, a width of 190 μm, and a depth of 70 μm. Hu [16] used a multiphase jet machining (MJM) method based on which a mixture of abrasives and water was accelerated by compressed air to remove material from substrates. Channel-, dimple-, and helical-groove-type surface textures were fabricated with the desired accuracy and dimensions using optimized MJM process parameters. Thus, Hu [17] used two optimized processes to improve the processing efficiency of masked MJM. 

Studies have also been conducted on AAJ processing without a mask. Ghobeity [18] presented processing model predictions and experimental data related to the presence or absence of a mask when processing a channel using abrasive micromachining on a glass substrate. It was confirmed that the prediction model was similar to the experimental result of processing a 400 µm deep channel at a distance of 20 mm without a mask using a 760 µm nozzle. Microchannels can be machined without masks using a micronozzle with an extremely short standoff distance (SOD) [19]. However, the study was limited by the fact that is was conducted only on a groove-shaped channel.

Our research activities have focused on the development of micro-AAJ machining methods for friction reduction without requiring a mask. Through this study, we want to show that such processing is possible without a mask using the micro-AAJ method. In addition, by comparing the friction coefficient of texturing under various conditions, the relative relationship between the surface shape and the friction coefficient can be identified. In this paper, we introduce our micro-AAJ machining system, describe the results obtained while machining with it, and measure the coefficient of friction of microtextures fabricated using it.

## 2. Microsurface Texture for Friction Reduction Using Micro-AAJ Machining

### 2.1. Micro-AAJ System Description

**Figure 2:**
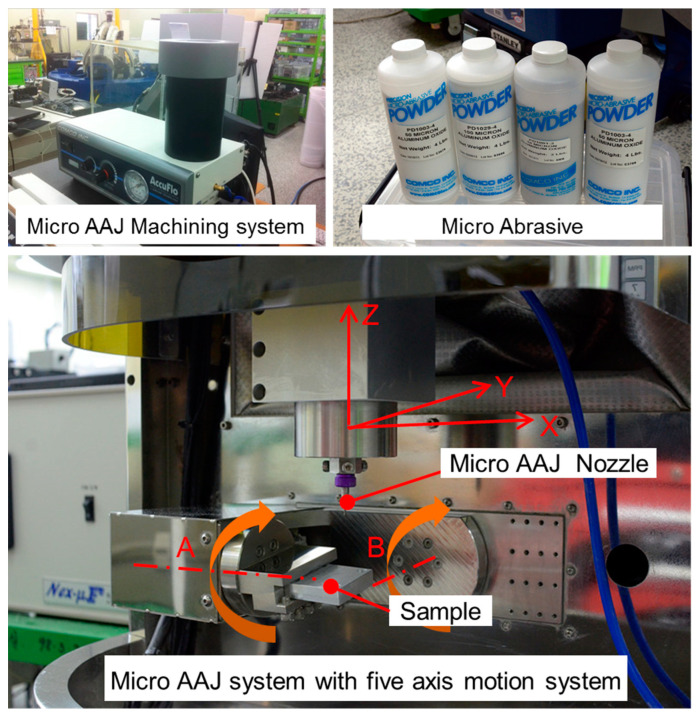
Micro-AAJ machining system including a five-axis positioning system.

Figure 2 shows the micro-AAJ machining system, which consists of a micro-AAJ nozzle and a five-axis positioning system. Various micro-AAJ nozzles can be used, depending on the required surface structure, and three-dimensional machining is possible. Abrasive particle powder was produced using an AccuFlo AAJ machine connected with a 6 bar pressurized air source. Figure 2 shows the positioning system, which moves along five axes. An X–Y–Z positioning system was also used for the micro-AAJ nozzle, consisting of a motorized positioning system for microaccuracy. An A–C rotation positioning system (A axis: 360°; B axis: 180°) was used to rotate the sample, consisting of a motorized rotation stage.

### 2.2. Micro-AAJ Machining

**Figure 3:**
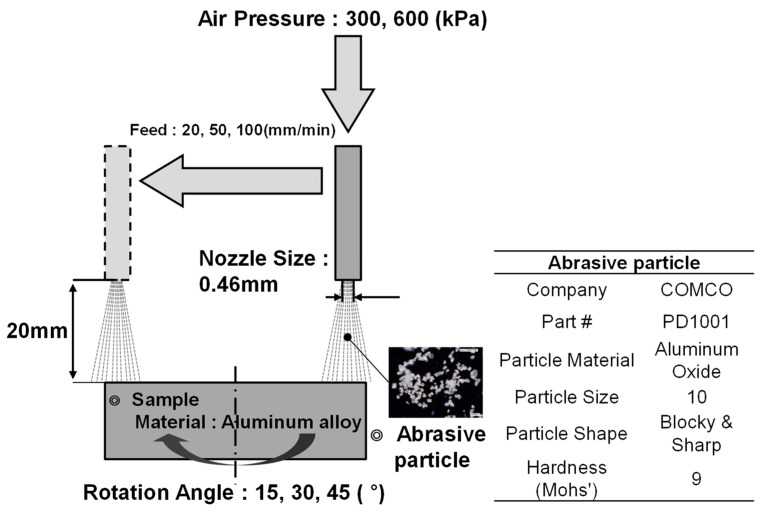
Microschematic diagram of the experimental machining of microsurface textures for friction reduction using the micro-AAJ machining system.

We used the setup shown schematically in Figure 3. The abrasive particles in the tank fell directly onto a vibrating feeder and were carried by the pressurized airflow into the tube. The abrasive particle powder consisted of 10 µm diameter alumina (Al_2_O_3_) sharp particles, which were injected into the compressed air flow to the nozzle through a rubber tube. This system allowed us to easily vary the feeding speed of the micro-AAJ nozzle and the rotation angle of the sample. The distance from the nozzle to the target could be varied within the range of 0 < d < 20 mm.

**Figure 4:**
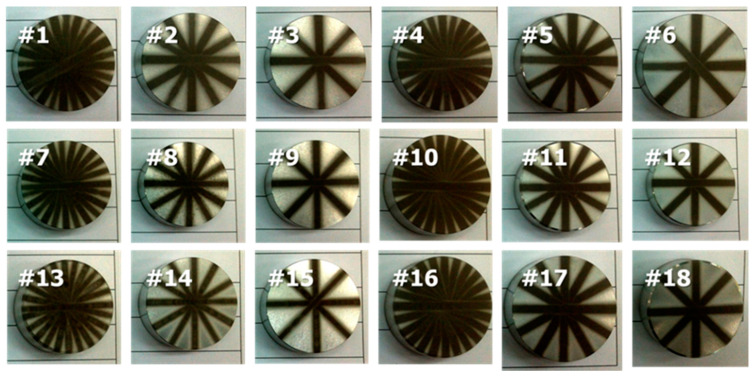
Images of samples machined using micro-AAJ.

**Figure 5:**
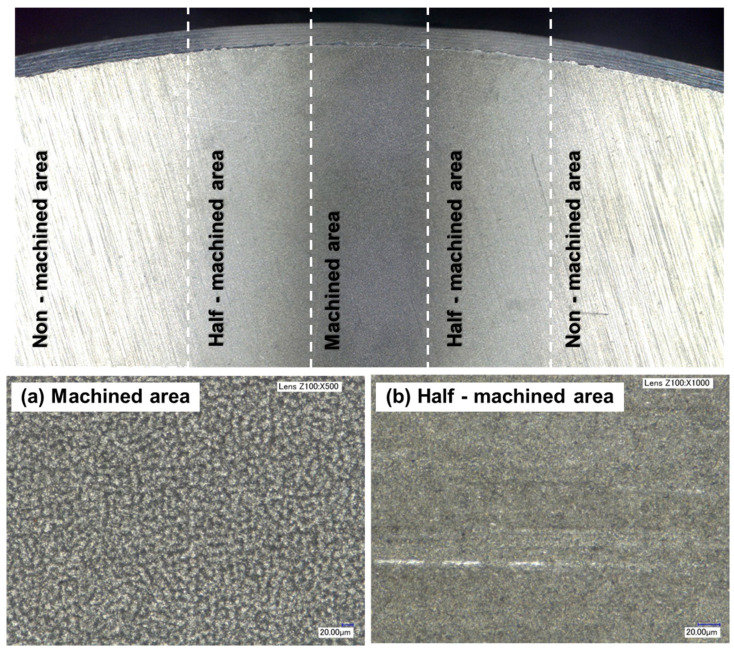
Optical microscopic images of the surface texture.

We tested ductile aluminum alloy, which is difficult to machine because of its low stiffness. We machined samples under eighteen different conditions, as listed in Table 1. Figure 4 shows images of machined samples. Figure 5 shows optical microscopic images of the surface texture. The surface can be divided into a non-machined area, a half-machined area, and a fully machined area. Figure 5a shows an area that was machined, and Figure 5b shows an area between the non-machined and fully machined areas. The size of the surface textures in the machined area was 20 µm. The width of each machined area varied according to the processing conditions.

**Figure 6:**
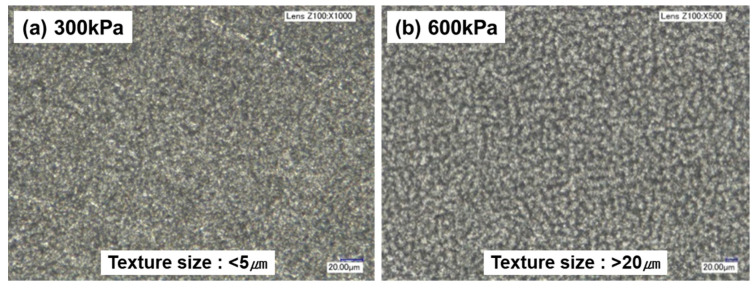
Optical microscopic images of surfaces that were machined at different pressures.

**Figure 7:**
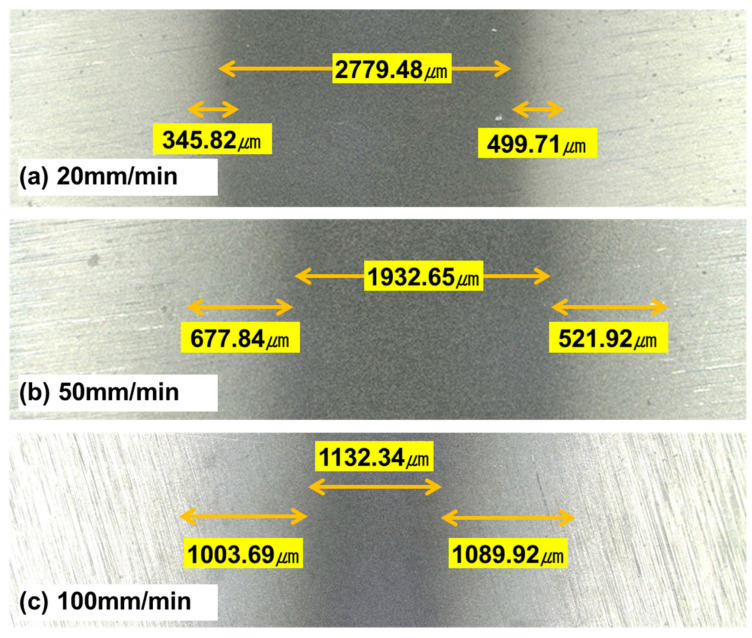
Optical microscopic images of surface textures machined by the micro-AAJ machining system at different traverse speeds.

Figure 6 shows optical microscopic images of surfaces that were machined at different pressures. We found that the size of the surface features was larger at 600 kPa than at 300 kPa. Figure 7 shows surfaces machined at different traverse speeds. As the traverse speed increased, the machined area decreased, and the half-machined area increased.

## 3. Experimental Setup

**Figure 8:**
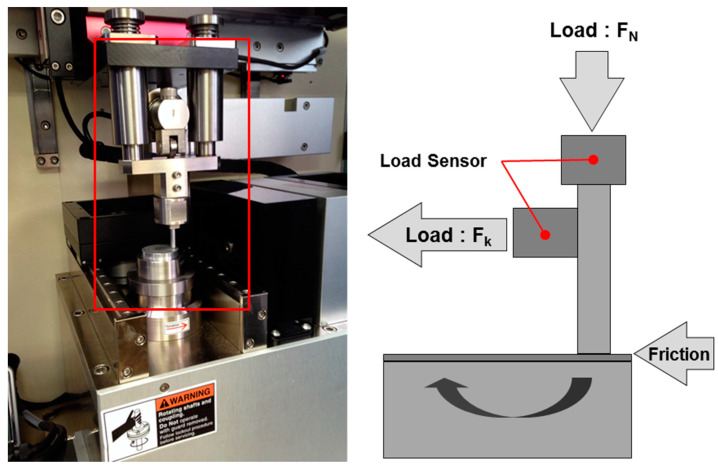
Friction test system and schematic image of the apparatus.

**Figure 9:**
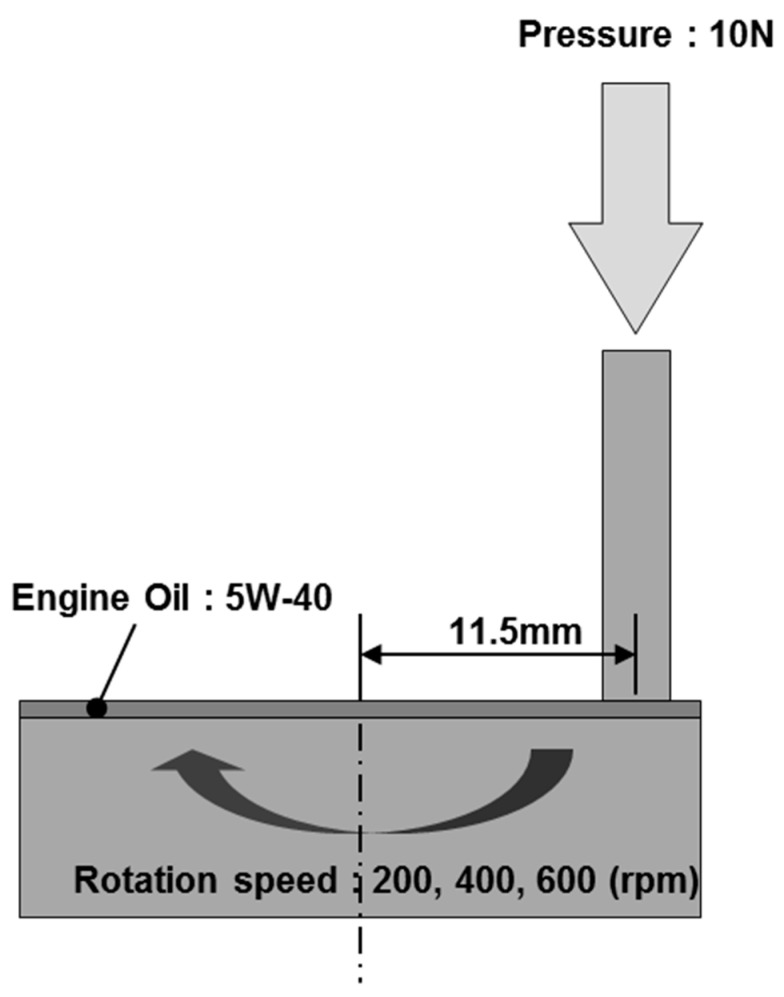
Schematic diagram of the experimental friction test.

The coefficient of friction was characterized using a pin-on-disk tester. Figure 8 shows the friction test system and a schematic diagram of the apparatus. The testing machine was designed for contact between a cylindrical pin and a planar surface. The pin was installed in a metallic holder and fixed such that it could not rotate. The pins were 3 mm in diameter and 5 mm in length. The center of the pin holder was loaded in the direction normal to the disk-shaped samples, which were located beneath the pins and were rotated. The test conditions are listed in Table 2, and the experiment is illustrated schematically in Figure 9. The sliding-contact zone was fully soaked in typical 5W40 engine oil, which acted as a lubricant. The temperature was maintained at 27 °C. The coefficient of friction was calculated using the frictional torque measured at different rotational speeds of the disk. The frictional radius was 11.5 mm. To avoid unstable initial conditions, each test was carried out after a breaking-in operation consisting of applying a normal load of 10 N and a rotation velocity of 200–600 rpm for a duration of 2 min.

## 4. Results and Discussion

In this section, we discuss the relationship between friction, rotation velocity, traverse speed, traverse-direction angle, and pressure of the micro-AAJ nozzle based on the experimental results.

**Figure 10:**
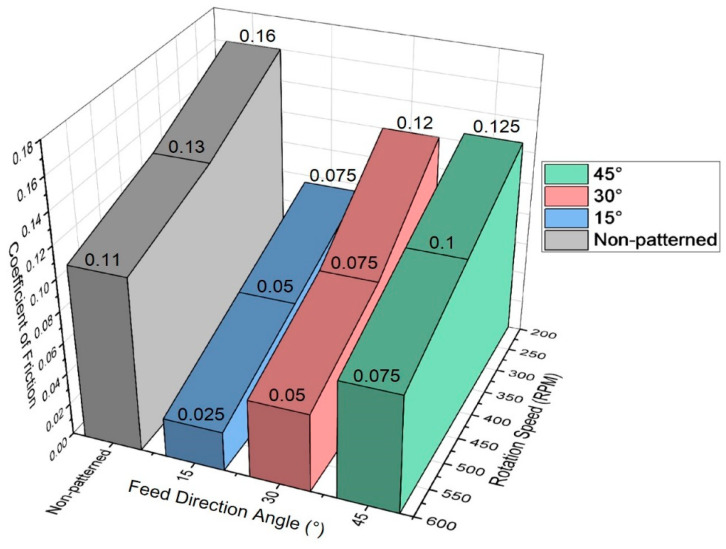
The relationship between the rotating velocity and friction coefficient for the sample.

The relationship between the rotation velocity and the coefficient of friction for the non-machined sample is shown in Figure 10. The coefficient of friction was measured at velocities of 200, 400, and 600 rpm. Typical boundary lubrication behavior was observed; an increase in the angular velocity resulted in a gradual decrease in the coefficient of friction. For the non-machined sample, the coefficient of friction (µ) at low angular velocities was µ ≈ 0.16. At angular velocities of 600 rpm, it was µ < 0.11. The trends of all of the textured sample curves were similar to the non-textured sample curve. However, the textured samples exhibited coefficients of friction that were lower than that of the non-textured sample. 

**Figure 11:**
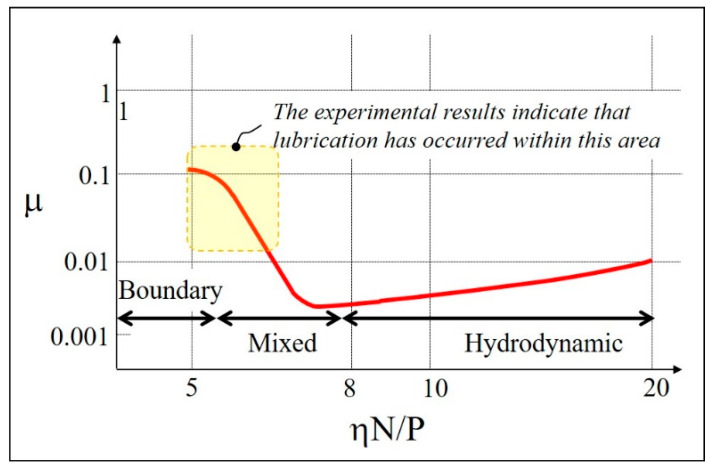
The relationship among the rotating velocity, friction coefficient, and lubrication regimes in the Stribeck curve of a journal bearing.

The tendency for the coefficient of friction to decrease with increasing angular velocity can be explained by the Stribeck diagram shown in Figure 11. Stribeck diagrams show the change in the coefficient of friction as a curve when switching from the fully hydrodynamic regime to the boundary lubrication regime [20,21]. The horizontal axis of the diagram is the value of Equation (1) presented below, and the vertical axis is the coefficient of friction μ value,

(1) $$\frac{\left(\eta \times N\right)}{P}$$

where η is the kinematic viscosity of the lubricant, N is the spindle speed (disk speed in this case), and P is the load.

According to Equation (1), when η and P are constant, the lubrication area changes from boundary lubrication to hydrodynamic lubrication as the value of N increases. As shown in Figure 11, the value of μ in the full lubrication regime shows a low value of less than 0.01, but as N increases, μ also increases. The region where μ decreases with increasing N is a mixed lubrication regime with μ values of less than 0.1. The µ values for the patterned samples identified in Figure 10 ranged from 0.025 to 0.125. That is, in the case of the samples patterned at 30° and 45° traverse-direction angles, the boundary lubrication regime transitioned to the mixed fluid lubrication regime as the disk rotation speed increased. When the transmission direction angle is 15°, the value of Equation (1) shifts to the right as the disk rotation speed increases within the mixed lubrication regime, and the coefficient of friction decreases. Therefore, it was confirmed that the friction test result according to the disk rotation speed of this experiment coincided with the trend shown in the Stribeck diagram.

**Figure 12:**
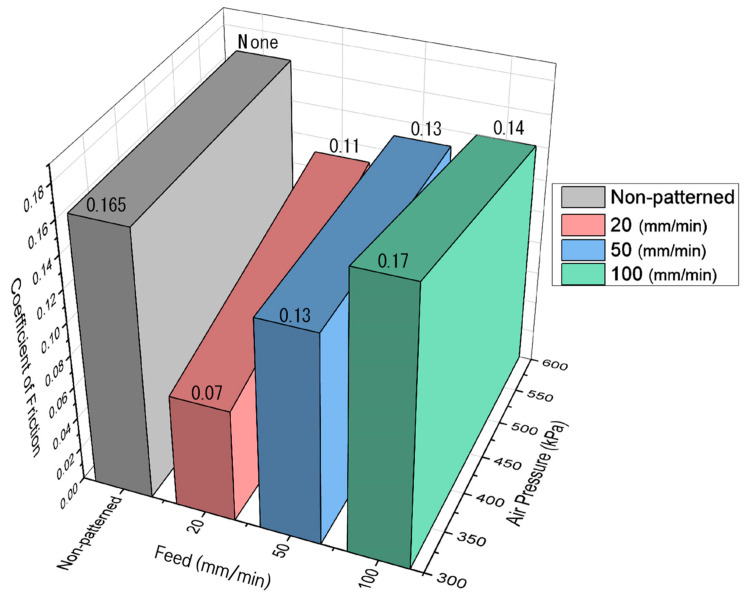
Coefficient of friction due to changes in traverse speed of the micro-AAJ nozzle during machining at 200 rpm.

**Figure 13:**
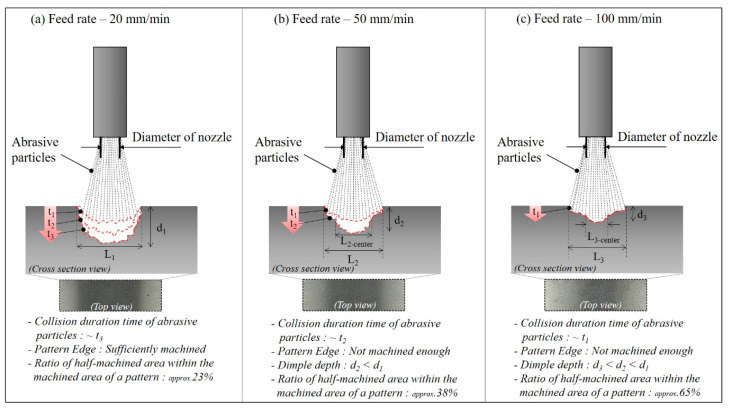
Pattern shape change mechanism according to the nozzle traverse speed of AAJ processing (at 300 Mpa).

Figure 12 shows µ as a function of the traverse speed of the micro-AAJ nozzle during machining. As the traverse speed of the micro-AAJ nozzle decreased and the density of the surface texture area increased, the friction also decreased. Figure 13 shows the shape and surface texture of the processing area that change according to the nozzle traverse speed. The traverse speed increases as the nozzle moves through the machined area at a relatively high speed. The impact time of abrasive particles is reduced, which can affect pattern depth and shape. Nouraei et al. [22] reported that in microchannel machining using abrasive air jets, the depth of the channel increases nonlinearly or linearly as the passing speed and jetting time of the abrasive particles increase, respectively. Park et al. [23] experimentally proved that the width of the channel increases nonlinearly and that the depth increases linearly as the number of nozzle scans increases. The pattern was processed using a masking film, but as the number of scans increased, the film was worn, and the width of the pattern increased because when the abrasive grains are ejected from the nozzle, they are also dispersed in the radial direction. As a result, abrasive particles impinge on the edge of the mask, and these particles repeatedly remove the edge surface as the number of scans increases. This means that even in maskless AAJ machining, increasing the abrasive impact time can sufficiently machine the pattern edge, reducing the ratio of the half-machined area to the total pattern area.

Li and Deng et al. [24] described that the number of particles colliding on the target surface decreases as the nozzle passing speed increases in AAJ micromachining. Furthermore, according to Li and Yeoh et al. [25], the change in the particle velocity in the radial direction of the nozzle increases as the axial distance from the nozzle outlet increases. That is, the velocity of the abrasive particles on the target surface decreases significantly from the center to the edge of the pattern. Therefore, the faster the nozzle moves, the wider the half-machined area because the edge area of the pattern cannot be sufficiently processed due to the reduction of the colliding particles. As shown in Figure 13, the proportion of the half-machined area increases from 23% to a maximum of 65% depending on the traverse speed under the same spray pressure and abrasive particle size conditions.

**Figure 14:**
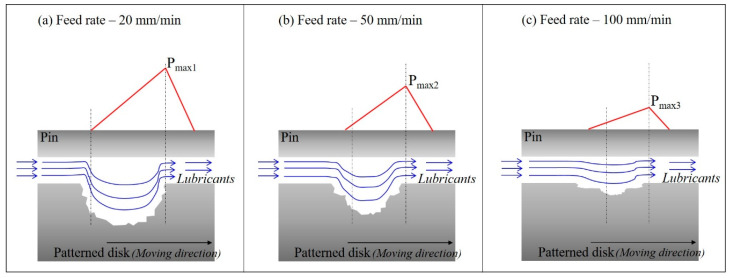
Mechanism showing the difference in the friction reduction effect according to the pattern change.

When the nozzle traverse speed is reduced, the pattern depth and width are increased, and the half-machined area is reduced, which can enhance the friction reduction effect of surface texturing. According to Li and Yao et al. [26], this is due to the increased surface texturing effect for the following reasons. First, the load-bearing capacity of the internal lubricant was improved by increasing the pattern depth and width. As shown in Figure 14, the deeper the pattern up to the critical depth, the larger the pressure difference between the pattern inlet and outlet. As a result, the load-carrying capacity (Pmax) of the lubricating oil is increased. Additionally, if the pattern area increases within a critical value, Pmax may occur continuously along the disk rotation direction. As a result, the load-bearing capacity of the lubricating oil is increased. Second, the increase in the pattern area and depth caused an increase in the lubricant storage area. The role of a lubricant reservoir is a well-known function of surface texturing technology, reducing friction in boundary lubrication or mixed lubrication regimes. Therefore, if the depth of the pattern is appropriately large, the lubricant storage capacity is increased, and the coefficient of friction is relatively reduced. Therefore, the friction is relatively reduced due to the increase in the pattern area and pattern depth.

**Figure 15:**
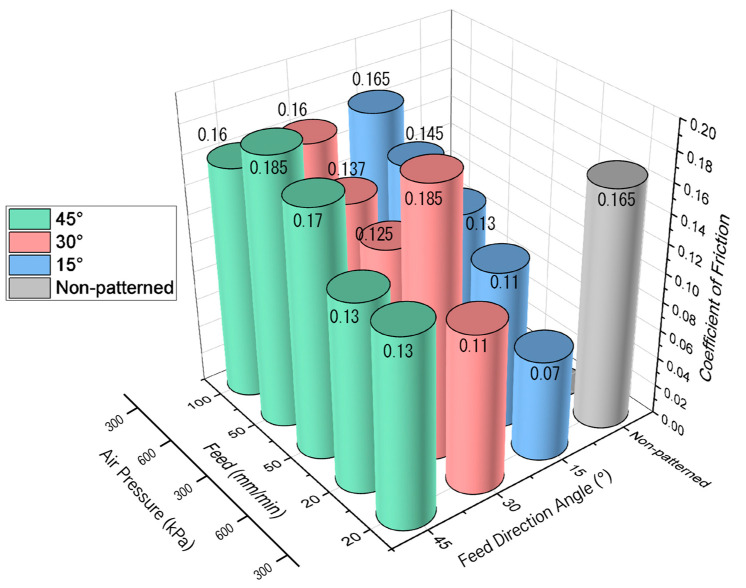
Coefficient of friction due to changes in traverse-direction angle of micro-AAJ machining at 200 rpm.

Figure 15 shows the change in the coefficient of friction due to changes in the traverse-direction angle of micro-AAJ machining. A small traverse-direction angle was used to increase the density of the textured area. As a result, the friction coefficients in the cases of 15 and 30° are lower than that at 45°. Specifically, as the traverse-direction angle decreased at a constant traverse speed of 20 mm/min, the frictional force decreased significantly, meaning that a friction-reducing effect can occur when there is enough of a patterned area on the contact surface. This result is consistent with the decrease in friction as the pattern area increases, as shown in the nozzle traverse speed experiment. Therefore, friction can be reduced by increasing the density of the pattern to a critical range by controlling the machining traverse-direction angle and nozzle traverse speed variables. Some of the coefficient values (#6, #9, and #12) are similar or somewhat higher than that of the non-patterned case, possibly because there are large, non-patterned areas in the 45 degree cases.

**Figure 16:**
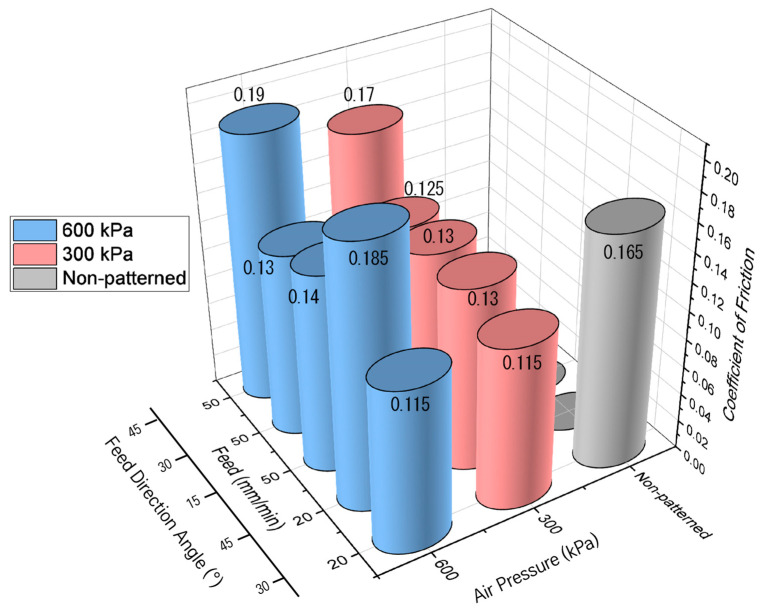
Coefficient of friction due to changes in the pressure of micro-AAJ at 200 rpm.

Figure 16 shows the coefficient of friction as a function of the pressure of the micro-AAJ. The coefficient of friction does not depend on the pressure change in the cases of 15 and 30°. The friction coefficients increase slightly with increasing pressure in the case of 45°, which can be explained by the fact that an increase in the pressure of micro-AAJ machining increases the surface roughness, which increases the coefficient of friction. However, if the contact area of the patterned surface is wide enough, little change in the friction occurs. A summary of the discussion is as follows.

- An increase rotating velocity results in a decrease in friction;
- A decrease traverse speed results in a decrease friction;
- An increase the density of the textured area results in a decrease in friction;
- An increase air pressure has little influence on the friction when the textured area is wide enough.

As a result, the best condition for reducing friction in micro-AAJ machining is as follows. The friction is minimized at a low traverse speed and wide patterned area under high-speed driving conditions. Experiment #1 satisfied these conditions, and the experimental results show the lowest coefficient of friction.

## 5. Conclusions

Our research activities have focused on the development of micro-AAJ machining methods for friction reduction without requiring a mask. We have described a micro-AAJ machining system and presented the obtained results while machining with it. We also measured the coefficient of friction of microtextured surfaces. The results of this work can be summarized as follows.

The coefficient of friction was measured at angular velocities of 200, 400, and 600 rpm. Typical friction behavior for boundary lubrication conditions was observed; an increase in the angular velocity resulted in a gradual decrease in the coefficient of friction. At very low velocities, µ ≈ 0.16; when the angular velocity was greater than 600 rpm, µ < 0.11.

The coefficient of friction depends on the traverse speed. As the traverse speed decreased, µ also decreased because the density of the surface texture was increased. A friction-reducing effect can occur when there is enough of a patterned area between the surfaces. The coefficient of friction does not depend on the pressure change if the contact area of the patterned surface is wide enough. Experiment #1 satisfied these conditions, and the experimental result shows the lowest coefficient of friction values.

This study showed that surface texturing processing is possible without a mask using the micro-AAJ method. By comparing the friction coefficient of texturing under various conditions, the relative relationship between the surface shape and the friction coefficient can be identified. We also presented an optimal solution verified by experiments.

Since the analysis of machined surface conditions such as surface roughness, surface morphology, and surface texture is important, additional research will be conducted on how these factors affect the frictional behavior of machined surfaces. We also plan to carry out additional research to enable industrial applications by identifying the relationship between surface texture and wear.

## Figures and Tables

**Table 1:** Micro-AAJ machining conditions.

No.	Traverse Speed (mm/min)	Air Pressure (kPa)	Angle (°)
#1	20	300	15
#2			30
#3			45
#4		600	15
#5			30
#6			45
#7	50	300	15
#8			30
#9			45
#10		600	15
#11			30
#12			45
#13	100	300	15
#14			30
#15			45
#16		600	15
#17			30
#18			45

**Table 2:** Friction test conditions.

Test Condition	
Pin	Aluminum alloy
Load (N)	10
Rotation velocity (rpm)	200, 400, 600
Rotation radius (mm)	11.5
Lubricant	Engine oil (5W40)
Temperature (°C)	27

## Data Availability

Not applicable.

## Nomenclature

µ	Coefficient of friction
d	Distance from the nozzle to the target
F_N_	Normal force
F_k_	Friction force
